# Unveiling Guyon’s Canal: Insights into Clinical Anatomy, Pathology, and Imaging

**DOI:** 10.3390/diagnostics15050592

**Published:** 2025-02-28

**Authors:** Sonal Saran, Pellauru Saavi Reddy, Kapil Shirodkar, Ankit B. Shah, Aakanksha Agarwal, Ankur Shah, Karthikeyan P. Iyengar, Rajesh Botchu

**Affiliations:** 1 Department of Diagnostic and Interventional Radiology, AIIMS Rishikesh, Rishikesh 249203, India; sonalsaranmalik@gmail.com; 2 Department of Musculoskeletal Radiology, Royal Orthopaedic Hospital, Birmingham B31 2AP, UK; saavireddy@gmail.com (P.S.R.); drkapilshirodkar@gmail.com (K.S.); 3 Eclat Imaging Centre Mumbai, Mumbai 400056, India; ankitbshah3581@gmail.com; 4 Department of Radio-Diagnosis, Mahatma Gandhi University of Science and Technology, Jaipur 302001, India; 5 Sadbhav Imaging Centre, Ahmedabad 380006, India; drankur203@gmail.com; 6 Department of Orthopaedics, Southport and Ormskrik Hospital, Mersey and West Lancashire Teaching NHS Trust, Southport PR8 6PN, UK; kartikp31@hotmail.com

**Keywords:** ganglion cyst, ulnar nerve entrapment at the wrist, MRI scan, diagnostic ultrasound, Guyon’s syndrome

## Abstract

Guyon’s canal, or the ulnar tunnel, is a critical anatomical structure at the wrist that houses the ulnar nerve and artery, making it susceptible to various pathological conditions. Pathologies affecting this canal include traumatic injuries, compressive neuropathies like ulnar tunnel syndrome, and space-occupying lesions such as ganglion cysts. Ulnar tunnel syndrome, characterised by numbness, tingling, and weakness in the ulnar nerve distribution, is a prevalent condition that can severely impair hand function. The canal’s intricate anatomy is defined by surrounding ligaments and bones, divided into three zones, each containing distinct neural structures. Variations, including aberrant muscles and vascular anomalies, can complicate diagnosis and treatment. Imaging techniques are essential for evaluating these conditions; ultrasound provides real-time, dynamic assessments, while magnetic resonance imaging (MRI) offers detailed visualisation of soft tissues and bony structures, aiding in pre-surgical documentation and pathology evaluation. This review article explores the anatomy, pathologies, and imaging modalities associated with Guyon’s canal and underscores the necessity of understanding Guyon’s canal’s anatomy and associated pathologies to improve diagnostic accuracy and management strategies. By integrating anatomical insights with advanced imaging techniques, clinicians can enhance patient outcomes and preserve hand function, emphasising the need for increased awareness and research in this often-neglected area of hand anatomy.

## 1. Introduction

Guyon’s canal is named after the French surgeon Jean Casimir Félix Guyon, who first described this anatomical structure in the late 19th century. Guyon made significant contributions to urology and anatomy, and his work included detailed studies of the wrist’s complex anatomy [1]. Guyon’s canal, or ulnar tunnel, is a narrow anatomical passage located at the wrist through which the ulnar nerve and artery travel as they enter the hand, making them susceptible to compression and injury [1,2]. Despite its critical role in hand function, Guyon’s canal often receives less attention in clinical practice and medical literature than other anatomical regions, such as the carpal tunnel. However, it is crucial for clinicians to understand its anatomy, potential pathologies, and the imaging modalities used to diagnose conditions affecting this region, particularly those specialising in hand surgery, neurology, and musculoskeletal radiology.

In recent years, increasing recognition of the clinical conditions related to Guyon’s canal pathology has spurred more detailed investigations into its anatomy, variations, and associated disorders. Pathologies of Guyon’s canal can range from traumatic injuries, such as fractures and dislocations, to compressive neuropathies and space-occupying lesions, such as ganglion cysts or tumours. Among these, ulnar nerve compression—commonly referred to as ulnar tunnel syndrome—is one of the most frequently encountered conditions. This syndrome can present with symptoms such as numbness, tingling, and weakness in ulnar nerve distribution, significantly impacting hand function and quality of life [3,4].

Understanding the clinical anatomy of Guyon’s canal is fundamental for diagnosing and treating conditions affecting the ulnar nerve and artery. The canal itself is bounded by osseous and ligamentous structures, and its configuration can vary depending on individual anatomical differences and pathological states. Awareness of anatomical variants, such as accessory muscles or vascular structures, is essential for both clinical evaluation and surgical planning, as these can influence the presentation of symptoms and the choice of treatment. Imaging plays a pivotal role in the assessment of Guyon’s canal pathologies. While clinical examination and electrophysiological studies are essential diagnostic tools, imaging provides direct visualisation of the canal’s structure and any pathological changes.

This review article aims to provide a comprehensive review of the clinical anatomy, pathology, and imaging of Guyon’s canal. By integrating anatomical knowledge with the latest advances in imaging, the goal is to enhance the understanding of how these pathologies present, progress, and can be effectively managed, thus improving patient outcomes and preserving hand function.

## 2. Clinical Anatomy

**Figure 1:**
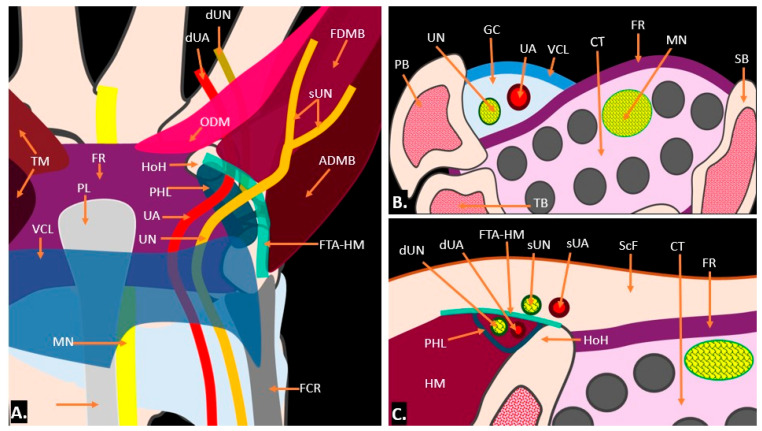
Illustration showing the anatomy of Guyon’s canal: (**A**) presents a frontal view in the coronal plane; (**B**,**C**) display axial sections at the proximal and distal levels, respectively. TM = thenar muscle; FR = flexor retinaculum; VCL = volar carpal ligament; MN = median nerve; PL = palmaris longus; UN = ulnar nerve; UA = ulnar artery; FCR = flexor carpi radialis; PHL = pisohamate ligament; HoH = hook of hamate; ODM = opponens digiti minimi; ADMB = abductor digiti minimi brevis; FDMB = flexor digiti minimi brevis; sUN = superficial branches of ulnar nerve; dUN = deep branches of ulnar nerve; dUA = deep branches of ulnar artery; FTA-HM = fibro-tendinous arch of hypothenar muscle; GC = Guyon’s canal; PB = pisiform bone; TB = triquetral bone; SB = scaphoid bone; HM = hypothenar muscles; ScF = subcutaneous fat; FR = flexor retinaculum; and, CT = carpal tunnel.

**Figure 2:**
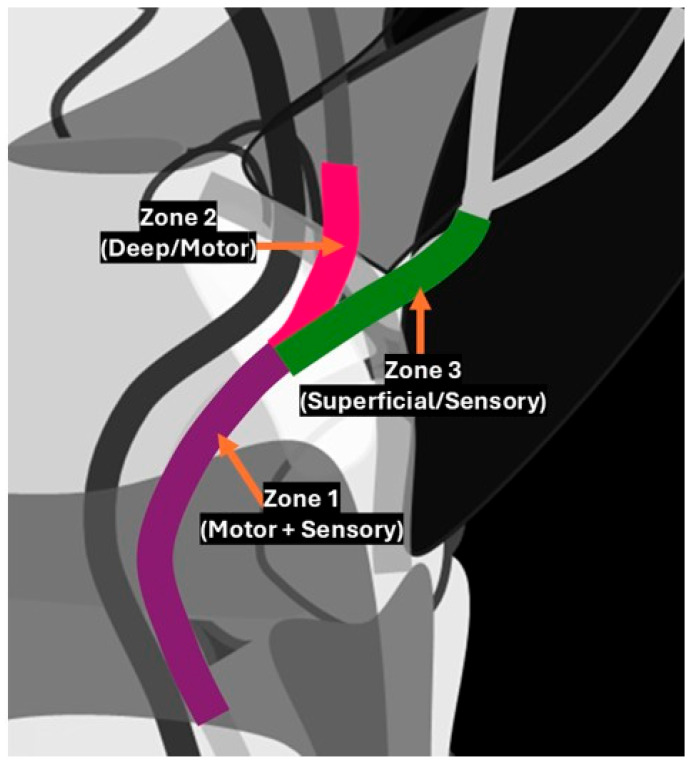
Illustration depicting the zonal anatomy of Guyon’s canal: Zone 1, located proximally, corresponds to both the motor and sensory components of the ulnar nerve; Zone 2 corresponds to the deep motor branch of the ulnar nerve, which innervates the intrinsic muscles of the hand; and Zone 3 corresponds to the superficial sensory branch, responsible for providing sensation to the ring and little fingers.

The boundaries of Guyon’s canal are defined by a complex arrangement of bones, ligaments, and muscles, creating a narrow passageway for the ulnar nerve and ulnar artery as they enter the hand. Medially, the canal is bordered by the pisiform bone, which acts as a key stabilising landmark. Laterally, the canal is bordered by the hook of the hamate, forming the outer edge of this tunnel. The roof of the canal comprises the superficial palmar carpal ligament (also known as the volar carpal ligament), which reinforces the canal’s structure and provides protection for the neurovascular contents. The floor of Guyon’s canal is formed by the transverse carpal ligament, also known as the flexor retinaculum, along with the deeper hypothenar muscles, including the abductor digiti minimi. Together, these anatomical boundaries create a narrow yet protective passage that allows the ulnar nerve and artery to supply the hand while also being vulnerable to compression, leading to potential clinical conditions such as ulnar tunnel syndrome (Figure 1). Anatomically, the canal is divided into three zones. “Zone 1, located proximally, contains both the ulnar nerve and ulnar artery as they enter the canal together; Zone 2 contains not only the motor branch of the ulnar nerve, which supplies the intrinsic muscles of the hand but also branches of the ulnar artery that supply the deep palmar structures, including the deep branch of the ulnar artery that often travels alongside the motor branch of the ulnar nerve as it supplies the deep muscles of the hand; and Zone 3, which primarily contains the sensory branch of the ulnar nerve for cutaneous innervation of the ring and little fingers, may also have smaller, superficial branches of the ulnar artery that contribute to the palmar arch” (Figure 2) [5].

The ulnar nerve innervates numerous motor and sensory structures in the hand. Its motor innervation includes several key muscles: the hypothenar muscles (abductor digiti minimi, flexor digiti minimi brevis, and opponens digiti minimi), the medial two lumbricals, adductor pollicis, the deep head of flexor pollicis brevis, and the interossei muscles. Sensory innervation by the ulnar nerve covers the skin over the medial aspect of the palm, the dorsum of the hand, and both the dorsal and palmar surfaces of the medial one-and-a-half fingers. This distribution enables the ulnar nerve to play a vital role in fine motor control and sensory perception on the ulnar side of the hand, particularly supporting the small muscles necessary for precise hand movements [5].

Several anatomical variations can be present in the canal, including aberrant muscles, vascular anomalies, and variations in the bifurcation of the ulnar nerve [6]. A detailed understanding of the clinical anatomy, complex structure, and potential variations of Guyon’s canal is essential for diagnosing and treating nerve compression syndromes and other pathologies.

## 3. Radiological Techniques and Anatomy

Imaging of Guyon’s canal primarily involves evaluating both bony structures and surrounding soft tissues, making it crucial for diagnosing conditions affecting this area. Conventional radiography is typically the first imaging technique used, providing information about fractures, especially those involving the hook of the hamate and calcific tendinopathy of the flexor carpi ulnaris [7]. Due to its superficial location, ultrasound imaging is highly effective for examining Guyon’s canal, offering dynamic, non-invasive, real-time imaging with excellent spatial resolution. This makes it valuable for detecting nerve compression and guiding interventions, such as aspiration of ganglion cysts or hydro-dissection for ulnar nerve entrapment. Although ultrasound imaging is operator-dependent, it is widely accessible and cost-efficient and allows for comparisons with the contralateral side [8].

Magnetic resonance imaging (MRI) is especially beneficial when pre-surgical documentation is required or for comprehensive three-dimensional evaluation of the pathology. MRI provides detailed imaging of both soft tissues and bones across a larger field of view but is more expensive, time-consuming, and less readily available [8]. Computed tomography (CT), while less useful for soft tissue assessment, excels in visualising bony structures and is particularly useful for detecting fractures of the hook of the hamate [9]. The choice of imaging modality depends on the clinical scenario, the suspected pathology, and the need for detailed anatomical visualisation.

### 3.1. Ultrasound Technique and Anatomy

**Figure 3:**
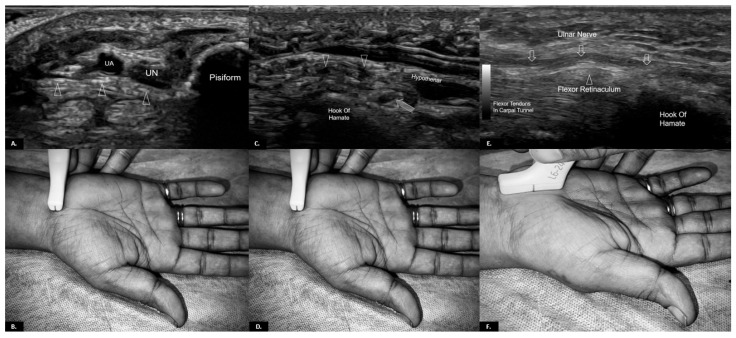
Ultrasound anatomy of Guyon’s canal with probe positioning: (**A**) the Axial Gray-scale Ultrasound image at the level of the proximal Guyon’s canal, at the level of the pisiform bone, containing the ulnar artery (UA) and ulnar nerve (UN), with its floor formed by the flexor retinaculum (open triangles); (**B**) probe positioning for (**A**); (**C**) Axial Gray-scale Ultrasound image at the level of the distal Guyon’s canal, at the level of the hook of the hamate, shows the ulnar nerve dividing into the sensory branch (open triangles) and deep motor branch (open arrow), with the deep motor branch travelling beneath the tendinous arch of the hypothenar muscles; (**D**) probe positioning for (**C**); (**E**) Longitudinal Gray-scale Ultrasound image of the Guyon’s canal shows the ulnar nerve (open arrows) and the underlying flexor retinaculum (an open triangle); and (**F**) probe positioning for (**E**).

For optimal visualisation of Guyon’s canal, the patient should be seated with the forearm resting on a flat surface, palm up, and the wrist slightly extended. A high-frequency linear transducer (10–18 MHz) is typically used to provide detailed images of the superficial structures of the wrist and hand (Figure 3) [10].

For a short axis view, begin the scan at the proximal wrist crease, placing the probe transversely over the volar aspect of the wrist. In this position, the ulnar nerve and artery are visualised as they enter Guyon’s canal. The ulnar nerve appears as a hypoechoic structure with a characteristic honeycomb pattern, while the ulnar artery appears as a round anechoic pulsating structure adjacent to it and shows a Doppler signal. As the scan progresses distally, assess the bifurcation of the ulnar nerve into its motor and sensory branches. This division typically occurs within the canal and is critical for identifying pathology affecting specific branches. It is important to identify the borders of the canal. The roof is formed by the volar carpal ligament, the floor by the transverse carpal ligament, and the canal’s radial and ulnar boundaries by the hook of the hamate and pisiform, respectively. The pisohamate ligament can also be seen extending from the pisiform to the hamate.

For a long-axis view, rotate the transducer 90 degrees and evaluate the ulnar nerve as it courses through Guyon’s canal. In this view, the ulnar nerve appears as a linear hypoechoic structure with fascicular pattern. This view can be helpful for assessing the continuity of the nerve and detecting any nerve thickening or oedema that may indicate compressive neuropathy. Careful scanning of the entire length of Guyon’s canal is necessary to identify any space-occupying lesions, such as ganglion cysts or tumours, that may compress the ulnar nerve. Variants such as accessory muscles or aberrant vessels should also be noted, as they can alter the clinical presentation and guide management.

### 3.2. MRI Technique and Anatomy

For optimal imaging of Guyon’s canal, the patient should lie semi-prone or prone, with the affected upper limb placed in a superman position entering the gantry or in a supine position with the arm by the side. A dedicated wrist coil or a small extremity coil is used to achieve high-resolution images of the wrist and hand. A comprehensive MRI protocol for Guyon’s canal includes a mixture of the following sequences: (1) axial T1/PD-weighted sequence for anatomical detail; (2) axial, coronal, and sagittal fluid-sensitive sequences (STIR, T2 or PD-weighted sequences with fat suppression) to identify pathology, highlighting areas of nerve oedema, inflammation, or cystic lesions; (3) gradient echo images for identifying any calcifications or subtle bone pathology, such as fractures; and, (4) post-contrast sequence (optional). Contrast-enhanced MRI (CE-MRI) is indicated in the evaluation of Guyon’s canal to enhance the assessment of various pathologies. It is particularly useful for differentiating between solid tumours and cystic lesions, such as ganglion cysts, that may compress the ulnar nerve. CE-MRI also aids in evaluating vascular structures, helping to identify conditions like aneurysms or thrombosis affecting the ulnar artery. Additionally, it provides critical information about ulnar nerve oedema, inflammation, or injury when standard MRI findings are inconclusive, making it valuable for pre-surgical planning and ensuring detailed anatomical visualisation [11].

The ulnar nerve appears as a low-signal-intensity structure on T1-weighted images and may appear hyperintense on T2-weighted images if there is oedema. The nerve is seen as it bifurcates into its motor and sensory branches within the canal. The ulnar artery is usually visible as a round structure with low signal intensity on T1/T2-weighted images. Post-contrast sequences can help evaluate arterial flow and detect any aneurysms or thrombosis. The volar carpal ligament forms the superficial boundary of Guyon’s canal and appears as a thin band of low signal intensity on both T1- and T2-weighted images. The transverse carpal ligament/flexor retinaculum, forming the floor of the canal, is a thicker, more prominent structure of low signal intensity on both T1 and T2 sequences. It is seen deeper in the axial images, bordering the canal’s dorsal side. The pisiform bone (medial) and the hook of the hamate (lateral) are bony landmarks that define the borders of Guyon’s canal. A high signal on a fluid-sensitive sequence often indicates marrow oedema, which replaces bright marrow with hypointensity in a T1-weighted sequence. The pisohamate ligament connects the pisiform to the hook of the hamate and is critical in defining the ulnar boundary of the canal. On MRI, it appears as a low-signal-intensity structure [11].

## 4. Pathological Conditions

Pathological conditions affecting Guyon’s canal include those specific to this region, such as calcific tendinopathy of the flexor carpi ulnaris tendon, hypothenar hammer syndrome (HHS), and accessory abductor digiti minimi, as well as conditions that can occur elsewhere in the body, such as ganglion cysts, neuromas, lipomas, and tenosynovial giant cell tumours.

### 4.1. Accessory Abductor Digiti Minimi

**Figure 4:**
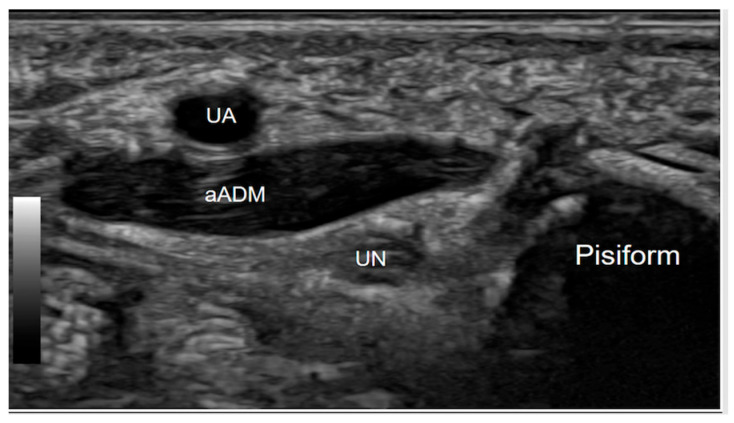
Axial Gray-scale Ultrasound image at the level of the proximal Guyon’s canal, at the level of the pisiform bone, demonstrates an anatomical variant where an accessory abductor digiti minimi muscle (aADM) overlies the canal. The ulnar nerve (UN) is present within the canal, while the ulnar artery (UA) is positioned volar to the accessory muscle.

This is an anatomical variant in the hand, where an extra muscle lies adjacent to the ulnar nerve as it passes through Guyon’s canal. This muscle variant can contribute to ulnar nerve compression within the canal, potentially leading to symptoms of ulnar neuropathy, such as numbness, tingling, or weakness in the ring and small fingers. Clinically, the presence of this muscle can complicate the diagnosis and management of ulnar nerve entrapment at Guyon’s canal, as it may mimic or exacerbate symptoms associated with other pathologies such as ganglion cysts or lipomas. The dynamic ultrasound assessment of this condition is particularly valuable. By having the patient actively abduct the fifth finger, real-time visualisation of the accessory abductor digiti minimi contraction and the ulnar nerve entrapment or compression at Guyon’s canal becomes possible. This dynamic evaluation is crucial for distinguishing between the asymptomatic presence of the accessory muscle and its pathological variant [12]. Identifying this variant on imaging or during surgical exploration is essential for appropriate management, as decompression or careful dissection around the muscle may be necessary to alleviate nerve compression symptoms effectively (Figure 4).

### 4.2. Hypothenar Hammer Syndrome (HHS)

**Figure 5:**
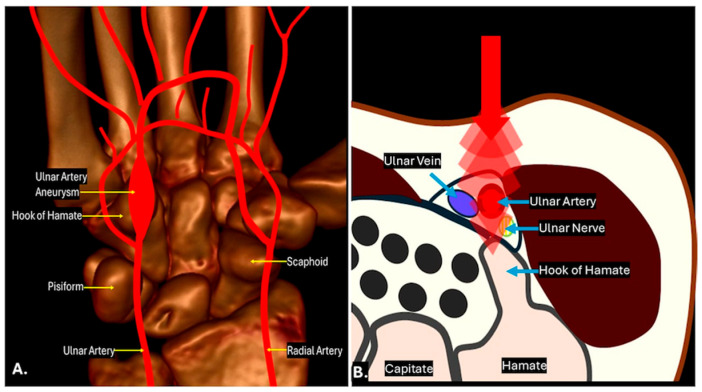
Schematic of hypothenar hammer syndrome with ulnar artery aneurysm shown in coronal (**A**) and axial plane (**B**).

This is a condition caused by repeated trauma or vibration to the hypothenar eminence, leading to damage of the ulnar artery and its branches, often within Guyon’s canal (Figure 5). The resulting vascular insufficiency can cause a range of symptoms, including cold intolerance, pallor, and claudication of the ulnar-sided digits, along with potential ulnar nerve compression. Clinically, it may present with hand weakness, sensory loss, or muscle atrophy due to the combined effect of arterial and nerve compromise. The diagnosis is often challenging, as the symptoms overlap with other conditions affecting the ulnar nerve in Guyon’s canal, such as ganglion cysts or accessory muscles. Early recognition and management, including avoiding further trauma and possible surgical decompression or vascular intervention, are crucial for preventing long-term functional impairment. Imaging plays a critical role in diagnosing HHS by assessing vascular integrity and identifying arterial occlusion, aneurysm formation, or thrombosis. Imaging techniques such as ultrasound and MRI are valuable tools for detecting vascular pathology and associated soft tissue changes. Colour Doppler ultrasound can detect arterial occlusion, segmental narrowing, thrombosis, or post-stenotic dilation of the ulnar artery, while turbulent flow patterns and aneurysm formation may be observed in areas of vascular damage. B-mode ultrasound further aids in identifying intimal thickening and mural thrombus, characteristic of HHS. MRI and MR angiography (MRA) provide detailed visualisation of soft tissue and vascular involvement, including adjacent nerve compression. MRA offers high-resolution imaging to assess stenosis, occlusion, aneurysm formation, and collateral vessel development in chronic cases. Additionally, MRI may reveal oedema and fibrosis in the hypothenar musculature, reflecting chronic vascular compromise. When ultrasound findings are inconclusive, contrast-enhanced MRA can be particularly useful for diagnosis and surgical planning.

### 4.3. Ganglion Cysts

Ganglion cysts are the most common space-occupying lesions occurring around the wrist joint. These benign, fluid-filled structures typically develop in association with tendon sheaths or joint capsules. When ganglion cysts form in or around Guyon’s canal, they can compress the ulnar nerve, leading to ulnar nerve entrapment and related symptoms such as tingling, numbness, or weakness in the hand [13,14,15].

**Figure 6:**
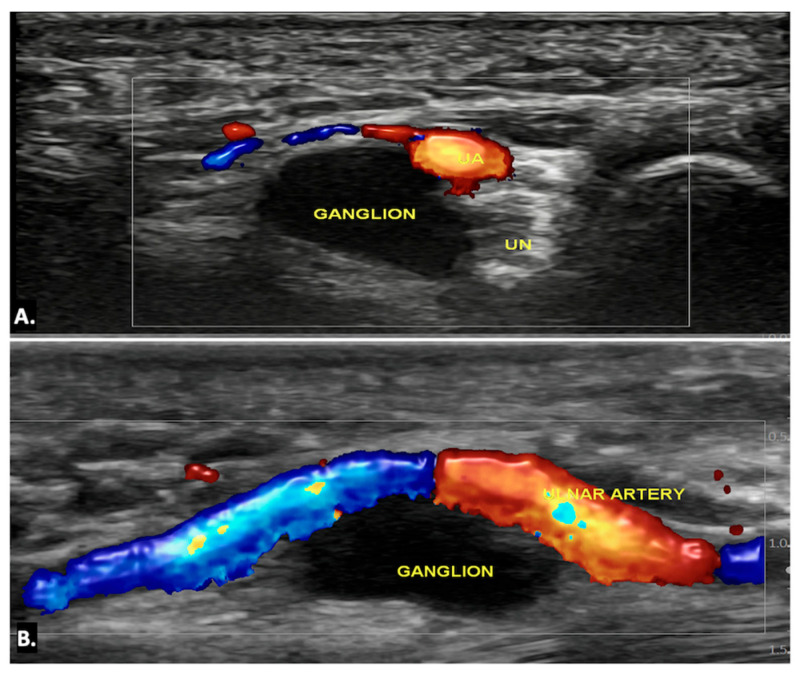
(**A**) Axial and (**B**) longitudinal colour Doppler ultrasound image showing an anechoic cystic lesion within Guyon’s canal, displacing the ulnar artery—likely a simple ganglion cyst.

Ultrasound imaging is often the first and typically the only imaging modality required to diagnose a ganglion cyst. On ultrasound imaging, ganglion cysts appear as well-circumscribed anechoic or hypoechoic lesions with posterior acoustic enhancement. They may also show communication with nearby joints or tendon sheaths, which helps confirm their origin. Doppler ultrasound is useful to exclude the possibility of a vascular lesion, such as an aneurysm, by demonstrating an absence of blood flow within the cyst. Additionally, internal signals within the lesion on Doppler may raise suspicion for a sinister aetiology, such as a sarcoma, warranting further investigation (Figure 6) (Video S1) [13,14].

**Figure 7:**
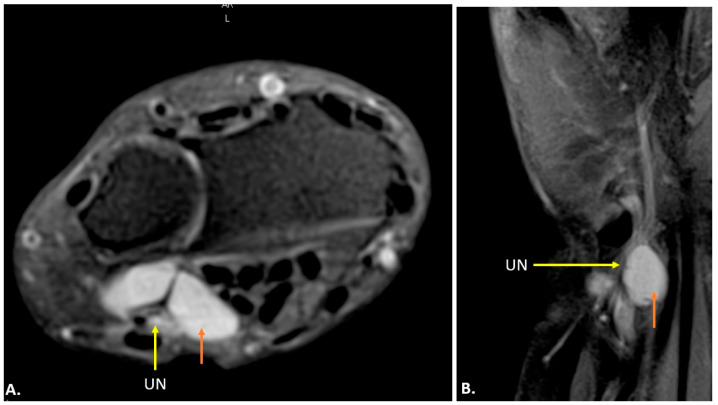
MRI reveals a well-defined, fluid-filled lesion within Guyon’s canal, consistent with a ganglion cyst (orange arrow), causing compression of the ulnar nerve (UN) (yellow arrow). Image (**A**) shows an axial view, while image (**B**) presents a long-axis view.

On MRI, ganglion cysts are well defined, hypointense on T1-weighted images, and hyperintense on T2-weighted images, reflecting their fluid content. Post-contrast images may show thin peripheral enhancement, but the cyst itself does not enhance. Unlike more complex cystic lesions, ganglion cysts usually do not contain calcific foci or haemorrhage. The cyst can cause displacement or compression of the ulnar nerve, which may show an increased signal on T2-weighted images, indicating oedema (Figure 7) [15].

**Figure 8:**
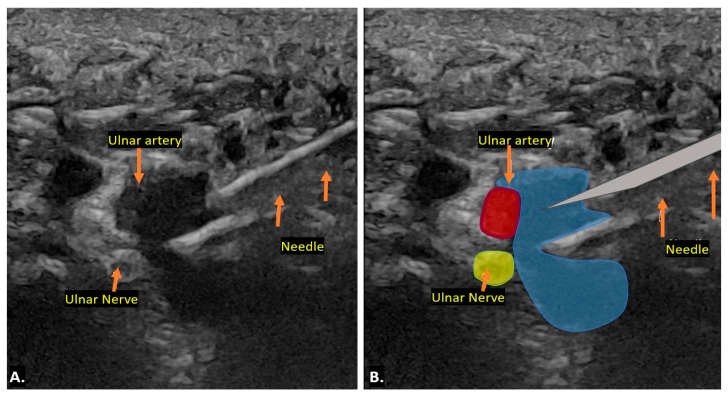
The image demonstrates ultrasound-guided aspiration of a ganglion cyst, with the needle carefully positioned within the cyst under real-time ultrasound visualisation to ensure precise targeting and safe fluid drainage (**A**). Graphic overlaying the ultrasound image (**B**).

Ultrasound imaging also plays a key role in the minimally invasive treatment of ganglion cysts. For aspiration, the patient is positioned similarly to the diagnostic ultrasound scan, and the area around the expected puncture site is cleaned with an antiseptic solution. An 18-gauge needle is often required, as the cyst contents tend to be thick and gelatinous. Despite multiple attempts, the contents may not be easily aspirated in some cases, requiring the clinician to repeatedly puncture the cyst wall to facilitate resolution. Even with successful aspiration, ganglion cysts are prone to recurrence. An intralesional steroid is often administered after aspiration to reduce the risk of recurrence. This approach provides effective symptom relief while minimising the need for surgical intervention (Figure 8) [16,17].

### 4.4. Neoplastic Conditions

A variety of neoplastic lesions can arise in the region of Guyon’s canal, ranging from benign to malignant tumours. Benign tumours commonly seen in this area include lipomas, haemangiomas, tenosynovial giant-cell tumours, and neurogenic tumours such as schwannomas or neurofibromas. Although malignant tumours are rare in this location, they can occur. One of the malignant lesions that may affect Guyon’s canal is synovial sarcoma, among other soft-tissue sarcomas. Malignant tumours in Guyon’s canal often present with more aggressive features, such as rapid growth and local invasion, and may lead to severe ulnar nerve dysfunction. Radiological imaging plays a crucial role in distinguishing between benign and malignant neoplasms, helping to guide diagnosis and subsequent treatment planning [8,18,19,20].

**Figure 9:**
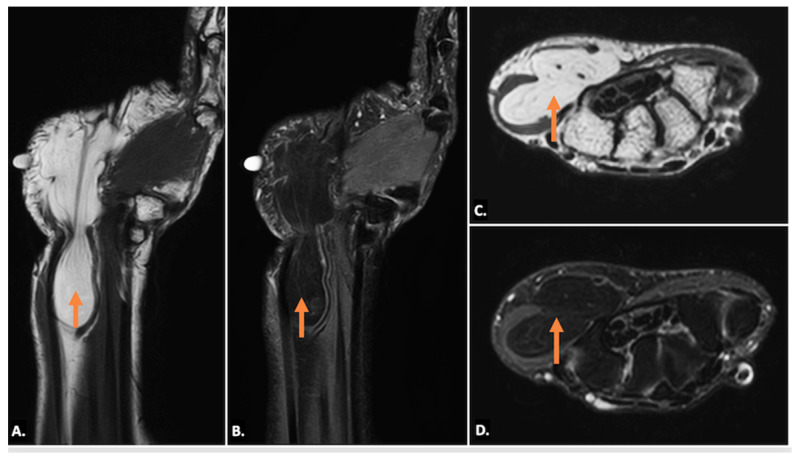
(**A**) T1-weighted coronal, (**B**) T1-weighted, fat-saturated coronal, and (**C**) T1-weighted axial and (**D**) T1-weighted fat-saturated axial MR images showing a typical lipoma (arrow) in Guyon’s canal, appearing as a T1-weighted hyperintense lesion (**A**,**C**) filling the Guyon’s canal with complete suppression of fat signals on T1-weighted, fat-saturated sequences (**B**,**D**).

**Figure 10:**
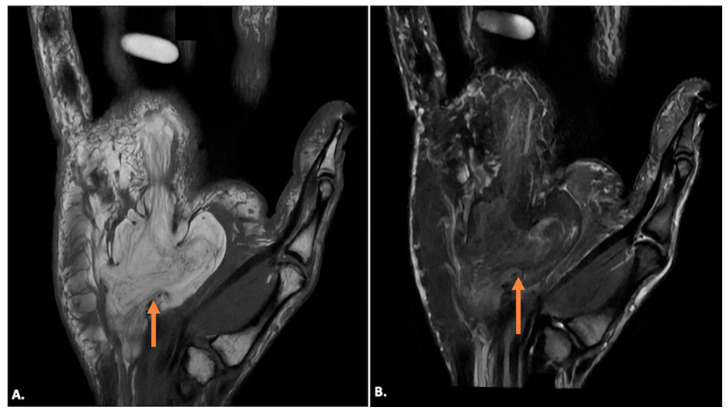
(**A**) T1-weighted coronal and (**B**) post-contrast T1-weighted, fat-suppressed coronal MR images showing an atypical lipomatous lesion (indicated by orange arrows) in Guyon’s canal, showing T1-weighted hyperintense lesion with hypointense septa in (**A**) with complete suppression of fat signals and enhancement of the septa in (**B**).

Lipomas appear as a well-defined, homogeneously iso-to-hypoechoic mass on ultrasound imaging corresponding to fat, and there is usually no internal vascularity on Doppler. It may have echogenic striations within the lesion. On MRI, lipomas are typically hyperintense on both T1- and T2-weighted images due to their high-fat content and demonstrate homogeneous fat suppression on fat-suppressed sequences (Figure 9). No internal enhancement is seen after gadolinium contrast administration. The presence of septa and nodules may point towards atypical lipomatous lesions (Figure 10) [18]. Hemangiomas present as hypoechoic or heterogeneous masses on ultrasound imaging, often containing anechoic areas corresponding to vascular channels. Doppler imaging will demonstrate significant internal vascularity with low-resistance arterial and venous flow patterns. On MRI, haemangiomas are hyperintense on T2-weighted images due to the high fluid content and show low signal intensity on T1-weighted images. Following contrast administration, they exhibit intense, heterogeneous enhancement.

**Figure 11:**
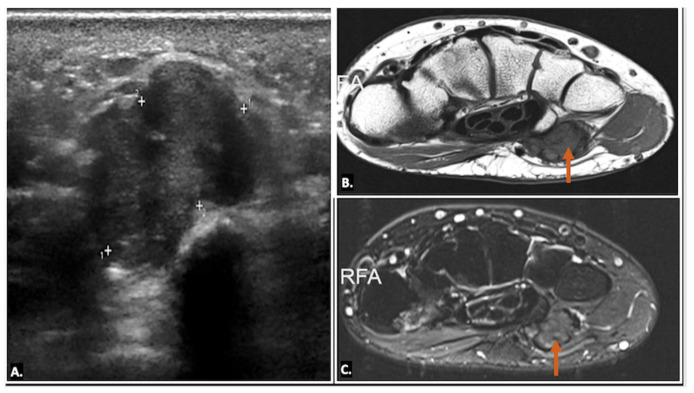
(**A**) Gray-scale Axial Ultrasound image showing a well-defined hypoechoic lesion in the region of Guyon’s canal (indicated by callipers). (**B**) T1-weighted and (**C**) T2-weighted axial MR images of the same case showing a well-defined lobulated lesion (arrow) appearing iso-to-hypointense to muscle on both (**B**) T1-weighted and (**C**) T2-weighted axial MR sequences in keeping with a tenosynovial giant-cell tumour.

**Figure 12:**
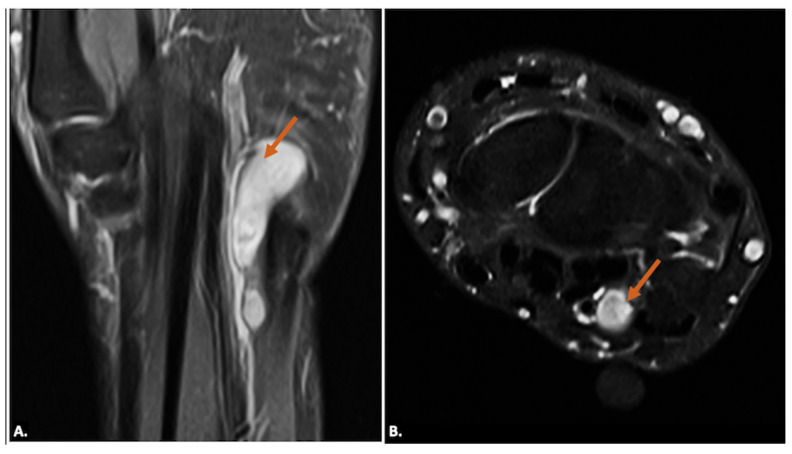
(**A**) T2-weighted, fat-suppressed coronal and (**B**) T2-weighted, fat-suppressed axial MR images of a neurogenic tumour showing a well-defined elongated lesion (arrow) along the course of the ulnar nerve appearing hyperintense on T2-weighted, fat-suppressed coronal (**A**) and demonstrating ‘Target-Sign’ with central hypointense signal on axial T2-weighted, fat-suppressed (**B**) sequences.

Tenosynovial giant-cell tumours generally arise in relation to the synovial sheath of the tendons or from the synovial lining of the joint. They appear as solid, hypoechoic masses on ultrasound imaging, and the lesions may show well-defined or ill-defined margins. On MRI, they typically appear as low-to-intermediate signal intensity on T1- and T2-weighted images due to hemosiderin deposition and may show blooming on gradient-echo sequences. Post-contrast images reveal variable enhancement (Figure 11). Neurogenic tumours appear as well-defined, hypoechoic masses along the nerve. Doppler imaging may show internal vascularity. On MRI, these lesions are isointense to hypointense on T1-weighted images and hyperintense on T2-weighted images, with a variable presence of “target” appearance on T2, and may show heterogeneous enhancement (Figure 12). The lesions are seen along the course of the ulnar nerve, with the nerve entering into and exiting from it [19].

Malignant tumours generally appear as heterogeneous, hypoechoic masses with irregular or poorly defined margins. They may show internal vascularity on Doppler imaging, often with high-flow signals. Synovial sarcomas are typically heterogeneous on both T1- and T2-weighted images, with areas of necrosis or haemorrhage. They may contain solid and cystic components. Post-contrast images demonstrate irregular, heterogeneous enhancement. Surrounding tissue involvement and local invasion may also be seen.

### 4.5. Flexor Carpi Ulnaris Calcific Tendinopathy

Calcific tendinopathy of the flexor carpi ulnaris tendon can present either as a chronic condition or, less commonly, as an acute inflammatory process known as acute calcific tendinitis. This condition is characterised by hydroxyapatite deposits within the tendon and can cause significant symptoms, including pain, swelling, and erythema. In some cases, the inflammation extends to surrounding tissues, potentially compressing the ulnar nerve, leading to ulnar neuropathy. Radiographs are often the first step in detecting calcific deposits within the tendon. In chronic cases, the calcifications appear more organised and denser, while in acute presentations, the calcium deposits may be less defined due to the associated inflammation and fluid buildup [7].

**Figure 13:**
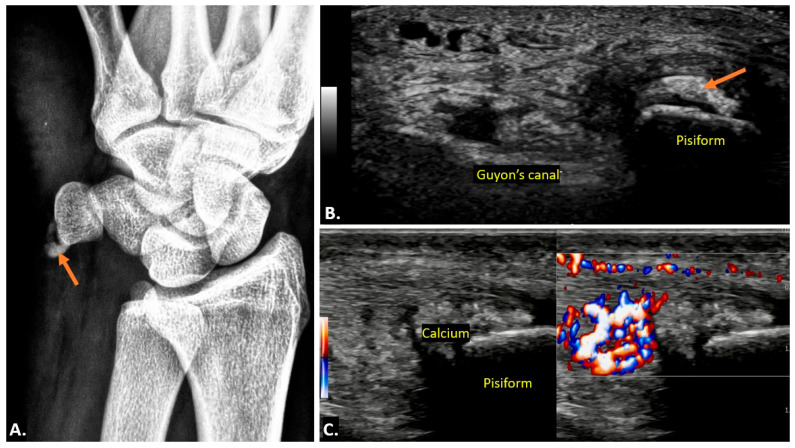
(**A**) Plain radiograph showing a radio-dense lesion located anterior to the pisiform bone. (**B**,**C**) Gray-scale Axial Ultrasound images of the same patient showing hyperechoic foci (orange arrow) at the flexor carpi ulnaris insertion over the pisiform bone, in keeping with flexor carpi ulnaris (FCU) calcific tendinopathy. (**C**) Gray-scale Longitudinal Ultrasound image showing calcium extending into the adjacent soft tissue. Ultrasound image with colour Doppler overlay demonstrates increased vascularity within the flexor carpi ulnaris (FCU) tendon and surrounding soft tissues, suggesting active inflammation.

On plain radiographs, calcific tendinopathy is typically seen as well-defined, amorphous, or cloud-like calcifications along the course of the flexor carpi ulnaris tendon near the pisiform bone. On ultrasound imaging, calcific deposits within the tendon appear as hyperechoic foci with posterior acoustic shadowing. These deposits are typically well-delineated in chronic cases. In acute calcific tendinitis, surrounding soft tissues may show signs of decreased echogenicity, indicating oedema and inflammation, and there may be associated fluid. Doppler ultrasound often shows increased vascularity surrounding the inflamed tendon and calcium deposits, consistent with acute inflammation (Figure 13) (Video S2). A synovial bursa is situated between the tendon and the pisiform bone. In some cases, patients may develop calcific bursitis. Ultrasound imaging is highly sensitive in detecting calcific deposits and assessing surrounding soft-tissue involvement, such as ulnar nerve inflammation or compression. Ultrasound imaging allows for dynamic assessment and is particularly useful in real-time evaluation and guidance for interventions such as aspiration [21,22].

On MRI, calcifications appear as low signal intensity on T1 images, with surrounding soft tissues showing intermediate signal intensity, representing oedema and inflammation. Calcific deposits also have low signal intensity on T2-weighted sequences, while surrounding inflamed soft tissues show a high signal. In cases of acute inflammation, there may be enhancement of the surrounding soft tissues and tendon sheaths due to increased vascularity and inflammation. This is particularly useful in assessing the involvement of nearby structures, such as the ulnar nerve, which, if compressed or inflamed, will show oedema with or without enhancement [21,22].

### 4.6. Idiopathic Ulnar Neuropathy

**Figure 14:**
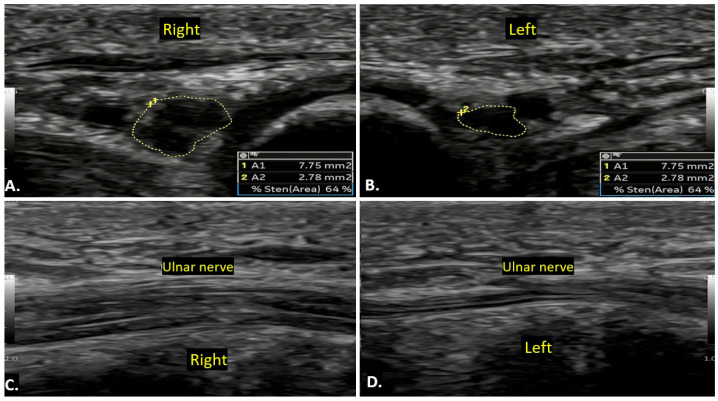
A case of idiopathic right ulnar neuropathy is illustrated. Image (**A**) shows an increased cross-sectional area of the right ulnar nerve compared to the left side (**B**), with a loss of the fascicular pattern. Image (**C**) presents a long-axis view of both wrists, highlighting the thickened right ulnar nerve in contrast to the normal left ulnar nerve (**D**). There is no evidence of a clear compressive cause.

Compression of the ulnar nerve in Guyon’s canal is second to the cubital tunnel. Sometimes, the neuropathy occurs in the absence of any identifiable cause. This condition involves dysfunction of the ulnar nerve without a clear compressive cause, leading to sensory and motor deficits in the ulnar nerve distribution. On ultrasound imaging, the ulnar nerve may appear thickened with an increased cross-sectional area as compared to the contralateral normal side, with effacement of normal fascicular architecture in the affected segment (Figure 14). No compressive mass is generally seen. On MRI, the ulnar nerve may show increased signal intensity on T2-weighted images, indicating nerve oedema or inflammation.

### 4.7. Vascular Conditions

**Figure 15:**
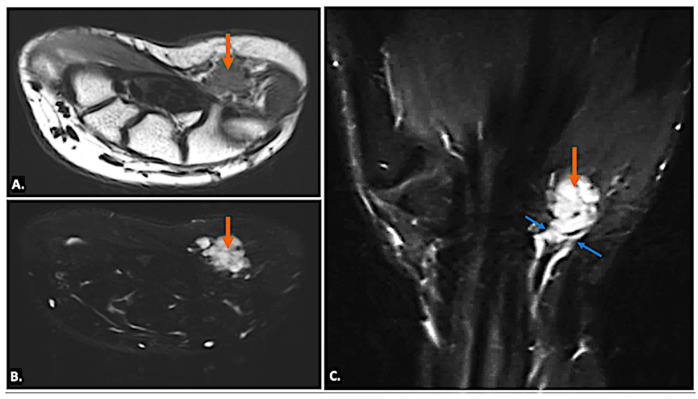
(**A**) T1-weighted axial, (**B**) T2-weighted, fat-suppressed axial, and (**C**) T2-weighted, fat-suppressed coronal MR images of an arteriovenous malformation (indicated by the orange arrow) in the region of Guyon’s canal. The lesion appears hypointense on the T1-weighted sequence (**A**), appears hyperintense on T2-weighted, fat-suppressed sequences, (**B**) and demonstrates continuity with vessels (blue arrows) (**C**).

**Figure 16:**
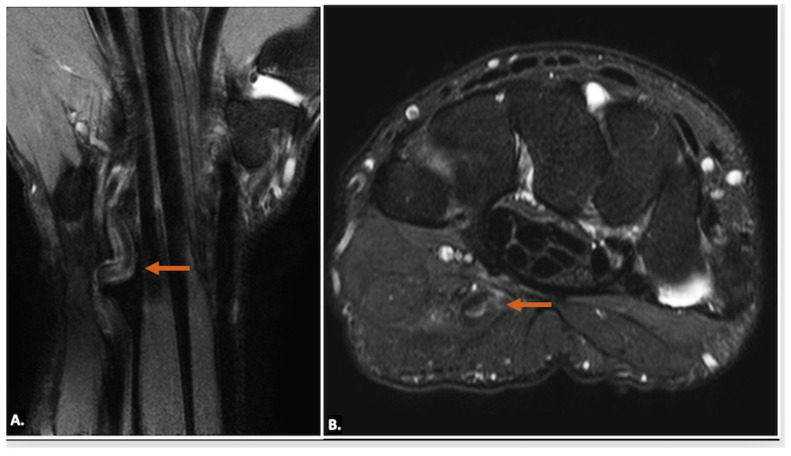
(**A**) T2-weighted, fat-suppressed coronal and (**B**) T2-weighted, fat-suppressed axial MR images reveal the tortuous irregular path of the ulnar artery (orange arrow) in the region of Guyon’s canal, resulting in ulnar neuropathy and causing neuropathic symptoms.

Other pathological conditions affecting Guyon’s canal include vascular abnormalities such as arteriovenous malformation (Figure 15), ulnar artery thrombosis, tortuosity (Figure 16), or aneurysms. Ultrasound imaging will show hypoechoic or anechoic mass with vascular flow seen on Doppler imaging in cases of aneurysm with absence of flow in a case of thrombosis. On MRI, thrombosis is seen as low or high signal intensity within the vessel lumen on both T1- and T2-weighted images, depending on duration. Aneurysms appear as well-circumscribed lesions, potentially with signal voids due to high flow. Pseudoaneurysm will show the presence of adjacent soft-tissue mass in close approximation with the lumen [23].

### 4.8. Traumatic Conditions and Miscellaneous Conditions

**Figure 17:**
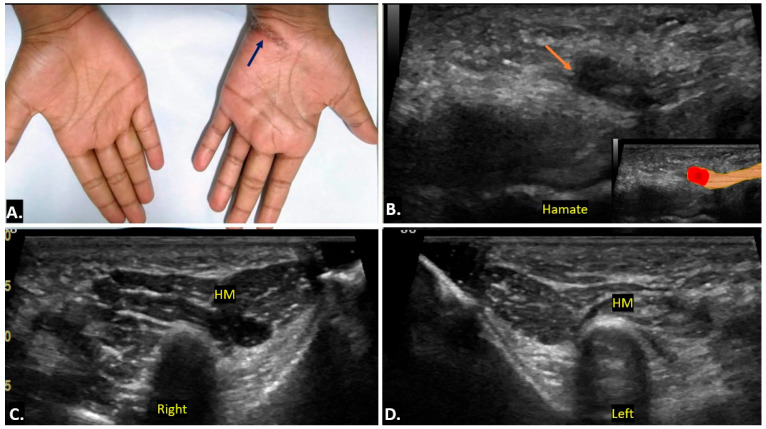
(**A**) Clinical photograph of a 20-year-old male patient presenting with a penetrating injury scar (purple arrow) in the region of Guyon’s canal with possible injury to the left-sided ulnar nerve; (**B**) Gray-scale Longitudinal Ultrasound image in the region of Guyon’s canal reveals a transected left-sided ulnar nerve with stump neuroma formation (orange arrow); and (**C**,**D**) Gray-scale Axial Ultrasound images show denervation atrophy of the left hypothenar muscles (HM) [D] compared to the contralateral normal right-sided muscles (**C**).

**Figure 18:**
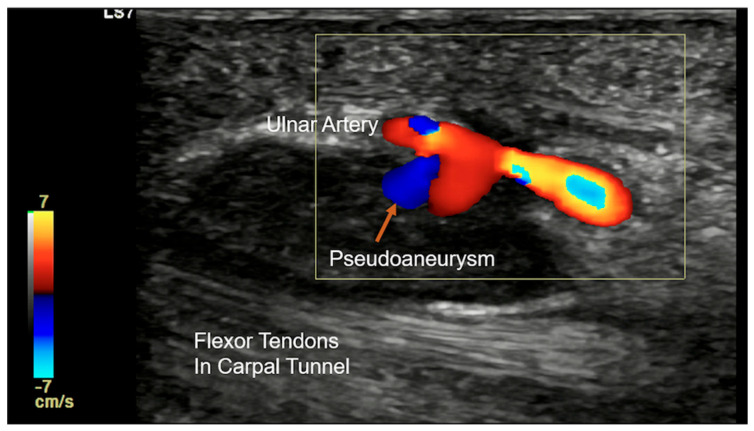
Colour Doppler longitudinal ultrasound image showing ulnar arterial pseudoaneurysm (orange arrow) in a case of hypothenar hammer syndrome.

Guyon’s canal can be affected by post-operative scar, rheumatoid arthritis causing pannus formation, or trauma-related lesions. Traumatic injuries in Guyon’s canal include both sharp and blunt trauma. Sharp injury is not uncommon and can result in Ulnar nerve transection and arterial injury. Depending upon the extent, the nerve may be partially or completely lacerated. Complete laceration can lead to the formation of stump neuroma, which appears to be a bulbous enlargement of the stump of the lacerated nerve (Figure 17). Blunt injury can involve bone as well as soft-tissue structures [24]. Hook of hamate fracture can occur as a result of a fall or in sports injury from racquets or bats while swinging. If occult on the standard radiograph, a carpal tunnel view will be able to demonstrate the fracture. CT and MRI can be considered if the radiograph is negative and there is a high suspicion of injury. Repeated trauma in this region in persons using a hammer/vibrating tool and in athletes is also seen (see Section 4.2). Repetitive trauma to the ulnar artery against the hook of the hamate can cause the formation of an aneurysm/pseudo-aneurysm or thrombosis (Figure 18) [25].

Compression at the inlet of Guyon’s canal, located proximal to the bifurcation of the ulnar nerve into its superficial and deep branches, can lead to both sensory and motor deficits. However, if the injury occurs more distally—such as from a hamate fracture or pisohamate ligament sprain—motor deficits may be observed without sensory involvement. A similar condition, known as handlebar neuropathy, results from prolonged compression of the ulnar nerve at the wrist, commonly seen in cyclists. This may cause focal swelling of the deep branch at the level of the hamate, along with atrophy or weakness of the dorsal interossei muscles. For treatment, an injection can be administered along the short axis of the ulnar nerve using an in-plane approach from the radial aspect within Guyon’s canal. To minimise the risk of iatrogenic injury, careful identification of the ulnar artery is crucial [26].

Summary of the most common pathologies of Guyon’s canal with the corresponding imaging findings are shown in Table 1.

## 5. Conclusions

In conclusion, understanding the anatomy, pathology, and imaging of Guyon’s canal is crucial for diagnosing and managing the ulnar nerve and vascular conditions at the wrist. This small but important anatomical region is susceptible to a variety of pathologies, including compressive neuropathies, space-occupying lesions, and traumatic injuries, all of which can have significant implications for hand function. Ultrasound and MRI play pivotal roles in diagnosing these conditions, offering detailed visualisation of soft tissues and bony structures. Comprehensive knowledge of the canal’s anatomy, potential anatomical variations, and the appropriate imaging techniques enable clinicians to provide targeted, effective treatment, ultimately improving patient outcomes.

## Figures and Tables

**Table 1:** Summary of the most common pathologies of Guyon’s canal with the corresponding imaging findings.

Pathological Condition	Ultrasound Findings	MRI Findings
Ganglion Cysts	A well-circumscribed anechoic or hypoechoic lesion with posterior acoustic enhancement. May show communication with joints or tendon sheaths. No internal vascularity on Doppler.	Well-defined, hypointense on T1 and hyperintense on T2-weighted images. Thin peripheral enhancement post-contrast. Possible nerve displacement/compression with T2 hyperintensity indicating oedema.
Lipoma	Well-defined, iso-to-hypoechoic mass, possibly with echogenic striations. No internal vascularity on Doppler.	Hyperintense on T1- and T2-weighted images. Homogeneous fat suppression. No post-contrast enhancement. Septa or nodules may indicate atypical lipomatous lesion.
Hemangioma	Hypoechoic or heterogeneous mass with anechoic areas (vascular channels). Doppler shows significant internal vascularity with low-resistance arterial and venous flow.	Hyperintense on T2, low signal on T1. Heterogeneous intense enhancement post-contrast.
Tenosynovial Giant-Cell Tumour	Solid, hypoechoic mass with increased vascularity on Doppler. May have well- or ill-defined margins.	Low-to-intermediate signal on T1/T2 due to hemosiderin. Blooming on gradient-echo sequences. Variable post-contrast enhancement.
Neurogenic Tumours (Schwannoma/Neurofibroma)	Well-defined, hypoechoic mass, sometimes with a “target” appearance (hypoechoic centre, hyperechoic rim). Minimal vascularity on Doppler. Follows ulnar nerve course.	Iso- to hypointense on T1, hyperintense on T2. May show “target” appearance. Heterogeneous enhancement. Follows ulnar nerve course.
Malignant Tumours (e.g., Synovial Sarcoma)	Heterogeneous, hypoechoic mass with irregular margins. Internal vascularity on Doppler with high-flow signals.	Heterogeneous signal on T1/T2 with necrotic or haemorrhagic areas. Irregular, heterogeneous enhancement post-contrast. Local invasion possible.
Flexor Carpi Ulnaris Calcific Tendinopathy	Hyperechoic foci with posterior acoustic shadowing. Chronic cases: well-delineated deposits. Acute cases: surrounding soft-tissue oedema and increased vascularity on Doppler.	Low signal calcifications on T1/T2. High signal in inflamed soft tissues on T2. Soft-tissue enhancement in acute cases.
Idiopathic Ulnar Neuropathy	Ulnar nerve swelling/thickening with loss of normal fascicular pattern. No compressive lesion.	Increased T2 signal in the affected nerve segment, indicating oedema or inflammation.
Vascular Abnormalities (Aneurysms, Thrombosis, AVM)	Hypoechoic/anechoic mass with vascular flow (aneurysms) or absent flow (thrombosis) on Doppler.	Thrombosis: Low or high signal in vessel lumen on T1/T2 (depending on stage). Aneurysms: Well-circumscribed lesion with potential signal voids from high flow.
Traumatic Injuries (Hook of Hamate Fracture, HHS, Nerve Laceration, Stump Neuroma)	Nerve laceration: Hypoechoic discontinuity with possible neuroma formation (bulbous enlargement). Fractures: May be occult on standard radiograph and on ultrasound. HHS: Vascular abnormalities on Doppler.	Fractures: Best seen on CT/MRI if radiographs are inconclusive. Neuroma: T2 hyperintensity with possible nerve thickening and discontinuity. HHS: Aneurysm/thrombosis findings.

## Data Availability

Data are available to share on request.

## Supplementary Materials

The following supporting information can be downloaded at: https://www.mdpi.com/article/10.3390/diagnostics15050592/s1, Video S1: Ultrasound video clip showing an anechoic ganglion cyst in Guyon’s canal causing neurovascular compression. Video S2: Ultrasound video clip showing calcific tendinopathy affecting Flexor Carpi Ulnaris.

## Abbreviations

MRI	Magnetic resonance imaging
CT	Computed tomography
STIR	Short Tau Inversion Recovery
PD	Proton density
HHS	Hypothenar hammer syndrome
